# The Role of Mycangial Fungi Associated with Ambrosia Beetles (*Euwallacea interjectus*) in Fig Wilt Disease: Dual Inoculation of *Fusarium kuroshium* and *Ceratocystis ficicola* Can Bring Fig Saplings to Early Symptom Development

**DOI:** 10.3390/microorganisms10101912

**Published:** 2022-09-27

**Authors:** Zi-Ru Jiang, Takeshige Morita, Shota Jikumaru, Keiko Kuroda, Hayato Masuya, Hisashi Kajimura

**Affiliations:** 1 Laboratory of Forest Protection, Graduate School of Bioagricultural Sciences, Nagoya University, Nagoya 464-8601, Japan; 2 Agricultural Technology Research Center, Fruit Tree Research Division, Hiroshima Prefectural Technology Research Institute, Hiroshima 739-2402, Japan; 3 Department of Plant Science, Graduate School of Agricultural Science, Kobe University, Kobe 657-8501, Japan; 4 Department of Forest Microbiology, Forestry and Forest Products Research Institute (FFPRI), Tsukuba 305-8687, Japan

**Keywords:** ambrosia *Fusarium* clade, *Ceratocystis* canker, insect-fungus symbiosis, *Neocosmospora metavorans*, tree pathogen, xylem dysfunction

## Abstract

The ambrosia beetle, *Euwallacea interjectus*, is a wood-boring pest and a vector of *Ceratocystis ficicola*, a pathogenic fungus causing fig (*Ficus carica*) wilt disease (FWD) in Japan. The ambrosia fungi, *Fusarium kuroshium* and *Neocosmospora metavorans*, have been frequently isolated from heads (including mycangia) of wild and reared adult female *E. interjectus*, respectively. However, the exact mechanisms driving FWD as well as the interactions between *F. kuroshium* and *C. ficicola* in fig orchard remain unclear. To verify the role of the mycangial fungi in the FWD progression, fig saplings were subjected to inoculation treatments (T1, *F. kuroshium*; T2, *N. metavorans*, reference positive control; T3, *C. ficicola*; T4, *F. kuroshium* + *C. ficicola*, realistic on-site combination). T3 and T4 saplings began wilting approximately 12 days after inoculation, leading to eventual death. Median duration from inoculation to death of the T4 saplings was approximately four days significantly faster than that of the T3 saplings. Xylem sap-conduction test indicated that dysfunction and necrosis area were considerably wider in the T4 saplings than in T3 saplings. These results demonstrate that the synergistic action of *F. kuroshium* and *C. ficicola* contributed to accelerated wilting in the saplings. Based on these discoveries, we proposed a model for system changes in the symbiosis between *E. interjectus* and its associated fungi in FWD in Japan.

## 1. Introduction

The pest fungus *Ceratocystis ficicola* Kajitani et Masuya is a soil-borne pathogen of fig trees (*Ficus carica* L.) in Japan [1,2,3,4]. Cultivation areas infested with fig wilt disease (FWD) were reported in 33/47 prefectures of Japan until 2017 [5]. This devastating epidemic of FWD has spread in various fig varieties including ‘Horaishi’ [4,6,7,8] and ‘Masui Dauphine’ [8,9,10]. Recently, in Greece, *C. ficicola* was found to cause serious disease in *F. carica* [11]. However, to date, the progression of FWD has not yet been fully elucidated.

The ambrosia beetle *Euwallacea interjectus* (Blandford) attacked poplar trees (*Populus deltoides* Bartram ex Marshall cv. ‘Deltoides’) in Argentina [12] and (*Populus* × *canadensis* Moench) in China [13] as well as box elder (*Acer negundo* L.) in the USA [14]. It is an important vector in FWD in Japan [4,6,15,16]. According to previous studies, *E. interjectus* may be a secondary pest of FWD because it does not contain *C. ficicola* within its mycangia (a special organ for storing and transporting fungal symbionts) [17,18]. Nonetheless, of the 22 species of ophiostomatoid fungi isolated from adult females of wild and reared *E. interjectus*, at least two dominant fungi, *Fusarium kuroshium* (Na, Carrillo et Eskalen ex Sand.-Den. et Crous) [18] and *Neocosmospora* *metavorans* (Al-Hatmi et al.) [19], respectively, may affect the progression of FWD. Morita et al. [7] reported that inoculation with *C. ficicola* killed the ‘Horaishi’ fig saplings. However, the degree of fungal symbionts of *E. interjectus* involved in FWD pathogenesis remains unclear. An understanding of the relationship between *C. ficicola* and symbionts, all of which are vectored by *E. interjectus*, may be useful for developing suitable disease control strategies.

Two ambrosia fungi of concern, *F. kuroshium* and *N. metavorans*, belong to the Ambrosia *Fusarium* Clade (AFC) [20,21]. *Fusarium kuroshium* was associated with *Euwallacea kuroshio* Gomez et Hulcr and shown to weaken the California sycamore (*Platanus racemose* Nutt.) and avocado tree (*Persea americana* Mill.) in California, USA [20]. *Neocosmospora* is a newly established genus in the *Fusarium solani* species complex (FSSC) (phylogenetic species 6) [22,23]. *Neocosmospora metavorans* has a wide range of plant hosts including avocado [24] and robusta coffee (*Coffea canephora* Pierre ex Froehner) [25]. Moreover, FSSC was primarily detected in discolored xylem and necrotic phloem from declining and defoliated Indian coral tree (*Erythrina variegata* L.) in Japan [26,27]. However, there have been no reports on the role of AFC in FWD. Detailed information is necessary to confirm the infection process of FWD and to determine whether *F. kuroshium* and *N. metavorans* are related to the decline and mortality of fig trees.

Back et al. [28] elaborated on the nature and outcome of interactions between soil-borne pathogens and plant-parasitic nematodes in terms of plant diseases. Numerous studies have demonstrated that co-inoculation with *Fusarium* fungi and nematode had a greater negative impact on plant growth and yields in winter wheat (*Triticum aestivum* L.) [29] and potato (*Solanum tuberosum* L.) [30]. However, no research has been conducted on the cooperative effects of soil-borne pathogens and ambrosia fungi in trees.

The objective of this study was to elucidate the individual and synergistic effects of soil-borne pathogens (*C. ficicola*) and wild *E. interjectus*–associated ambrosia fungus (*F. kuroshium*) in the symptom development of FWD through inoculation experiments including rearing *E. interjectus*-associated ambrosia fungus (*N. metavorans*). Hence, our goal was to assess the damage potential of *F. kuroshium* and/or *C. ficicola* to the fig saplings.

## 2. Materials and Methods

### 2.1. Inoculation Test

A total of 108 *F. carica* saplings (‘Masui Dauphine’; 2 years old; diameter 2–4 cm) were used in this study (Table 1). All saplings were commercial products and were brought from the nursery stock base to the greenhouse of Nagoya University (Nagoya, Japan) on 26 February 2019. The next day, they were transferred to plastic pots filled with gardening soil (Super soil, Akimoto Tensanbutsu Co. Ltd., Mie, Japan) and watered every few days. Two weeks after transplanting, each pot was covered with a fine nylon net to prevent entry of root feeders (scarabaeid beetle). After the germination of new shoots, the saplings were retained in the greenhouse until required for the inoculation test.

To relieve the problems associated with high temperatures during the growth period of the fig saplings, nylon sunshade curtains were installed inside the greenhouse. To monitor the temperature, a button-type temperature datalogger (Thermochron G type, KN laboratories Inc., Osaka, Japan) was set at a height of approximately 1.2 m above the floor. The maximum air temperature in the greenhouse was 37.75 ± 2.93 °C (mean ± SD) during the experimental period from 29 July to 4 September 2019.

**Figure 1:**
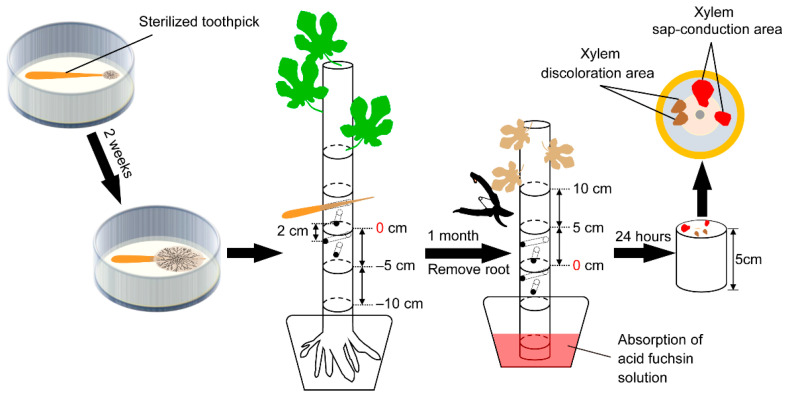
The layout of the inoculation site of the fungi tested on the fig sapling and observation of the cut end of the sapling after dye injection.

**Figure 2:**
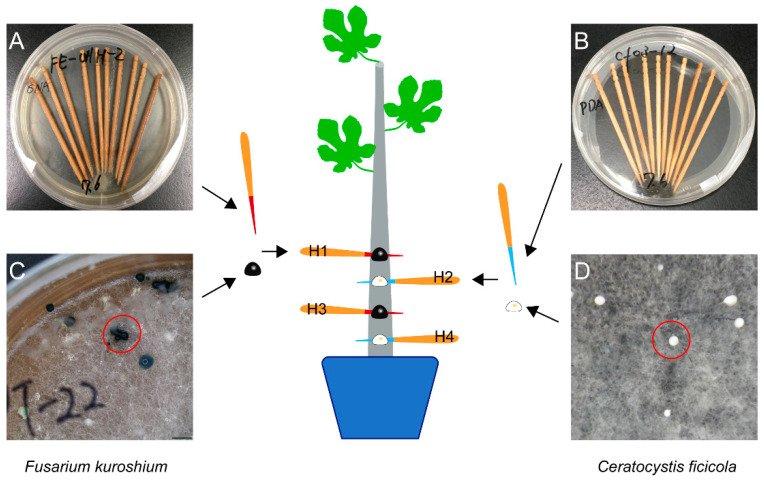
The dual inoculation of both *Fusarium kuroshium* (H1 and H3) and *Ceratocystis ficicola* (H2 and H4) on the fig saplings. (**A**) Toothpicks with hyphae of *F. kuroshium*; (**B**) toothpicks with hyphae of *C. ficicola*; (**C**) *F. kuroshium* with the greenish conidial masses (red circle); (**D**) *C. ficicola* with buff-yellow ascospore masses (red circle).

From the Hiroshima Prefecture in Western Japan, *F. kuroshium* (W1-h, isolated from the head of an adult female wild *E. interjectus*), *N. metavorans* (R14-h, isolated from that of reared *E. interjectus*), and *C. ficicola* (cf-03, isolated from the soil surrounding the FWD tree) were obtained for use in an inoculation test (Table 1). Before inoculation, *F. kuroshium* and *N. metavorans* were grown on synthetic low-nutrient agar (SNA: 1 g KH_2_PO_4_; 1 g KNO_3_; 0.5 g MgSO_4_·7H_2_O; 0.5 g KCl; 0.2 g glucose; 20 g agar; 1 L distilled water) and *C. ficicola* was grown on potato dextrose agar (PDA: 4 g potato starch; 20 g dextrose; 15 g agar; 0.1 g streptomycin sulfate; 1 L distilled water) in a 9 cm Petri dish for 2–3 weeks (25 °C, dark). Simultaneously, ten sterilized toothpicks (L = 7 cm, d = 2.2 mm) were added to each dish to adhere to the hyphae of *F. kuroshium*, *N. metavorans*, and *C. ficicola* (Figure 1 and Figure 2). In addition, *F. kuroshium* with greenish conidial masses on SNA [20,31] and *C. ficicola* with buff-yellow ascospore masses on PDA [2] were prepared after 1–2 months of incubation (Figure 2). Sterilized toothpicks were also prepared as the control inocula (Table 1).

The fungal inoculation test was performed according to the method described by Morita et al. [7] (Figure 1). Before inoculation, a set of four vertical holes were made on each fig sapling (i.e., H1, H2, H3, and H4) by boring through the center of its stem (diameter = 4 mm) 8–14 cm above ground level with an electric drill. Prepared inoculation toothpicks of *F. kuroshium* (T1), *N. metavorans* (T2), and *C. ficicola* (T3) were inserted into the holes of 18 saplings.

For the dual inoculation of *F. kuroshium* and *C. ficicola* (T4), the toothpicks were inserted alternately: *F. kuroshium* into H1 and H3, and *C. ficicola* into H2 and H4 (Figure 2). As the control inoculum, only sterilized toothpicks (ST) were inserted into the holes of 18 saplings. Dual inoculation of *N. metavorans* + *C. ficicola* was not considered as *N. metavorans* is derived from reared (indoor) *E. interjectus* and does not coexist with *C. ficicola* in fig orchard (outdoor). After inoculation of T1–T4 and ST, the section of the toothpick protruding out of the hole was cut, and the inoculation site was covered with sealing film to prevent drying out. In addition, 18 unwounded fig saplings (CT) were included in this test. Symptom development in all 108 saplings was observed daily during the experimental period.

### 2.2. Classification of Wilt Symptoms

According to the results of Morita et al. [7], the external FWD symptoms in inoculated fig saplings were classified as follows (Figure S1): (1) no external symptoms (NS), no apparent difference compared to healthy CT saplings; (2) leaf wilt (LW), some leaves began to droop and wilt, but were still alive; (3) branch discolored (BD), all leaves became brown with branches discolored in the xylem; and (4) shoot sprout (SS), one or two shoots sprouted at the inoculation site after death of the upper branches.

### 2.3. Xylem Sap-Conduction Test

Sapling samples from each of the inoculation treatments (T1, T2, T3, and T4) were reaped four weeks post-inoculation, and their internal FWD symptoms were examined and compared to those of the control (ST and CT) (X; Figure S2).

To evaluate the water function in the main stems of the saplings, a dye injection was performed (Figure 1). Immediately after severing the main stem base, the ends of the cut stems were placed in 1% (*w*/*v*) aqueous acid fuchsin for 24 h in a greenhouse. Subsequently, the immersed stems were cut into 5 cm long segments (Figure 1). On the cut surfaces, the xylem sap-conduction area (pink area; functional xylem) dyed with acid fuchsin, xylem discoloration area (brown area; non-functional xylem) infested with fungal inocula, and the entire cross-sectional area (excluding the pith) were observed (Figures S3 and S4). RGB images were acquired using a Sony ILCE-6000 digital camera (Sony Corporation, Tokyo, Japan), and ImageJ software (Win64, version 1.52p, National Institutes of Health, Bethesda, MD, USA) was used to quantify the images.

The rate of the xylem sap-conduction area or xylem discoloration area (%) in each cross-section segment of the tested fig saplings was calculated as follows:

(1) pink or brown area/whole area ×100 %

### 2.4. Re-Isolation of Inoculated Fungi

The remaining saplings (T1, T2, T3, T4, and ST) were processed for the fungal re-isolation test (R; Figure S2).

Stems of the saplings were cut into 5 cm long segments, which were then separated between the top 1 cm and the bottom 4 cm. The top segment was divided into four parts (slices), and surface sterilized for 1 min in 70% (*v*/*v*) ethyl alcohol solution. The second sterilization was performed using 1% (*v*/*v*) metformin solution for 1 min. Finally, the samples were rinsed with sterile distilled water for 1 min and dried on sterile filter paper for 1 min. Subsequently, two of the four sterilized slices were placed on PDA (25 °C, dark). The inoculated fungi were confirmed by colony morphology analysis on PDA. The remaining two sterilized slices were stored in zippered plastic bags (Uni-pack^®^ A-4, Seisannipponsha Ltd., Tokyo, Japan) at approximately 25 °C for the detection of *C. ficicola* [7].

The re-isolation rate (%) was calculated as follows:

(2) NF/NS ×100 %

where NF is the number of slices from which the particular fungal species were re-isolated, and NS is the total number of slices used.

### 2.5. Statistical Analysis

Statistical analyses were performed using SPSS version 19.0 software (IBM Corporation, Armonk, NY, USA, 2010) and R 4.0.3 (R Core Team, Vienna, Austria, 2019). The duration from inoculation to death of the fig saplings (BD and SS) was compared between T3 and T4 using the Mann–Whitney *U* test. Curve fitting was used to examine the correlation between the re-isolation rate (*y*-axis) and distance from the inoculation site (*x*-axis) as well as calculate the value of the coefficient of determination (*R*^2^).

## 3. Results

### 3.1. Symptom Development

**Figure 3:**
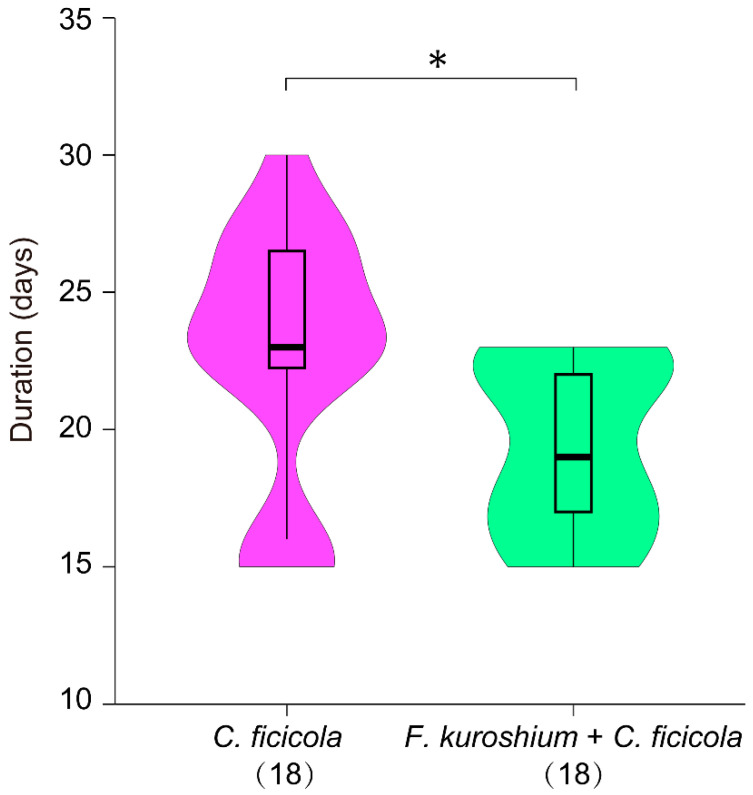
Violin plot representing the distribution in duration from the inoculation to death of fig saplings for T3 treatment (*Ceratocystis ficicola*) and T4 treatment (*Fusarium kuroshium + C. ficicola*) in the inoculation test. Boxes span the first to third quartiles; thick horizontal lines within the boxes show the median values; whiskers indicate values within the 1.5 interquartile range. The figure in parentheses indicates the number of saplings. *: statistically significant at *p* = 0.011 < 0.05 using the Mann–Whitney *U* test.

Approximately 12 days after inoculation, the T3 and T4 saplings exhibited typical wilting as an initial, external symptom (T3-09 and T4-14; Figures S1 and S2). Subsequently, all T3 and T4 saplings died within 30 days of inoculation; however, symptom development was not synchronous between T3 and T4 (Figure S2). The median duration from inoculation to death (taken as the date of the last sapling death) in T4 was approximately four (seven) days significantly faster than that in T3 (Figure 3), which indicates the synergistic effect of the dual isolates (T4: *F. kuroshium* + *C. ficicola*). In contrast, no symptoms were observed on T1, T2, ST, and CT (Figure S2). There were some SS saplings (T3: 15/18; T4: 10/18; Figures S1 and S2), which seemed to have barely survived in part under the inoculation site.

### 3.2. Xylem Sap-Conduction

In a total of 54 fig saplings (nine saplings each of T1, T2, T3, T4, ST, and CT; Figure S2), the functional xylem area was visualized by the absorption of the acid fuchsin solution from the ends of their cut stems (Figures S3 and S4).

**Figure 4:**
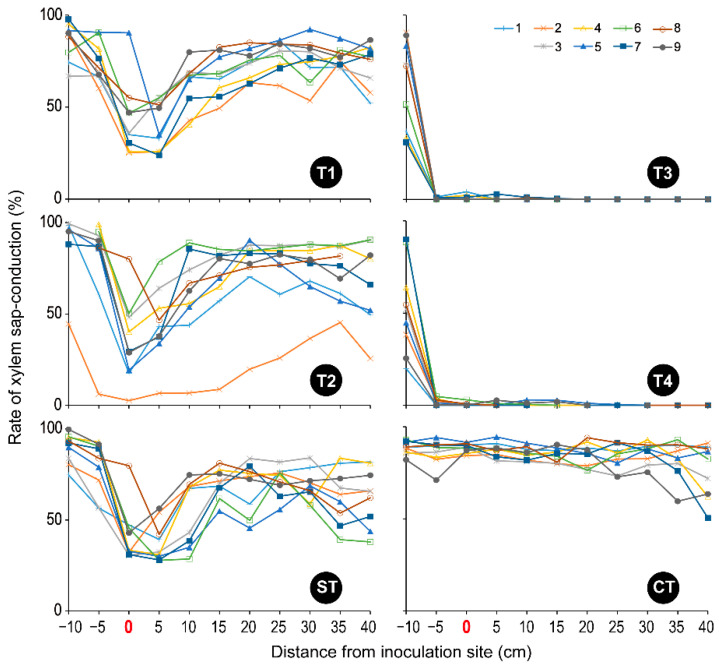
The rate of xylem sap-conduction in each fig sapling tested. T1: *F. kuroshium*; T2: *N. metavorans*; T3: *C. ficicola*; T4: *F. kuroshium* + *C. ficicola*; ST: sterilized toothpicks; CT: none. The treatment information is shown in Table 1.

**Figure 5:**
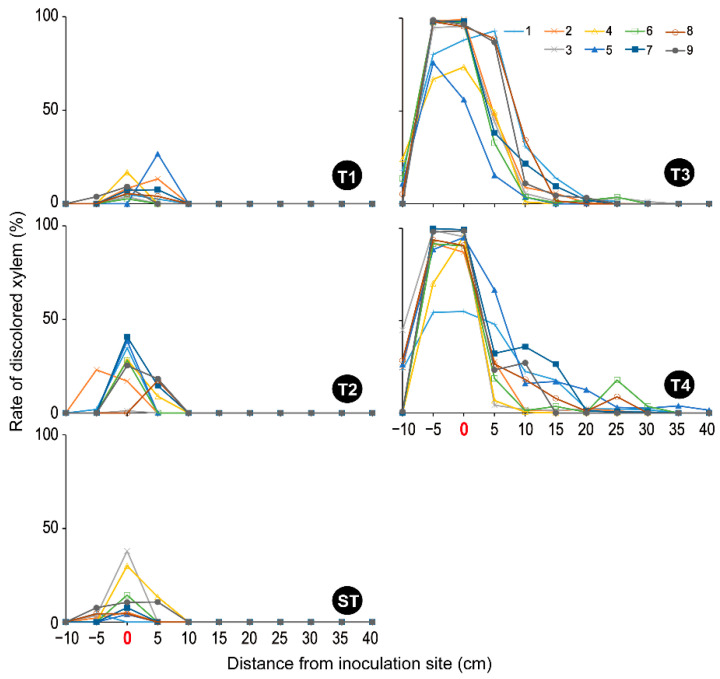
The rate of discolored xylem in each fig sapling tested. T1: *F. kuroshium*; T2: *N. metavorans*; T3: *C. ficicola*; T4: *F. kuroshium* + *C. ficicola*; ST: sterilized toothpicks; CT: none. The treatment information is shown in Table 1.

In CT, from the base to the top of the stem, the entire xylem of almost all segments was dyed pink, except for the pith (Figures S3 and S4), which maintained more than 80% of the sap-conduction area (Figure 4). The prepared fig saplings were healthy and their growing conditions were appropriate. In T1, T2, and ST, at 0–10 cm from the inoculation site, some parts of the wound xylem were unstained with dye solution (Figures S3 and S4), and their sap conduction was reduced (Figure 4). The fig sapling slices appeared brown, especially at the inoculation site (0 cm of the distance) (Figure S3), where the maximum rate of the xylem discoloration area of each sapling was 2.59–26.66% in T1, 1.16–40.72% in T2, and 4.54–37.94% in ST, respectively (Figure 5). However, in these three treatments, the entire xylem of other segments (−5 cm or less and 15 cm or more of the distance) was dyed pink, as in CT (Figure S3), and showed a high rate of sap-conduction area (Figure 4) excluding one sapling (T2-02; No. 2 in T2). This finding indicates that water flowed from the roots to the upper stems, passing through the narrow but still functional zones of xylem in the area up to 5 cm above the inoculation site.

In contrast, all T3 and T4 xylems were unstained throughout the region from 5 cm below to 40 cm above the inoculation site (Figure S3), thus showing almost 0% of the sap-conduction area (Figure 4). In addition, the upper segments appeared drier. Both xylem discoloration areas were centered between 5 cm above and below the inoculation site but extended widely (−10 cm to 30 cm and −10 cm to 40 cm of the distance in T3 and T4, respectively) (Figure S3 and Figure 5). Treatments T3 and T4 clearly discolored the saplings more intensely than the T1 and T2 treatments (Figure S3 and Figure 5). As evidence of this, the maximum rate of xylem discoloration in each sapling was 54.43–99.83% in T4 and 73.68–99.03% in T3 (Figure 5).

### 3.3. Re-Isolation of Inoculated Fungi

A total of 1362 isolates were obtained from the 45 fig saplings (nine saplings of T1, T2, T3, T4, and ST; Figure S2). *Fusarium kuroshium* (36 isolates) and *N. metavorans* (64 isolates) were detected in T1 and T2, respectively. *Ceratocystis ficicola* in T3 and T4, and *F. kuroshium* in T4 did not appear. This result for *C. ficicola* was different from that reported by Morita et al. [7], perhaps because the sapling segments were too dry for the *C. ficicola* growth in our experiment. As expected, in the ST treatment, three inoculated fungi (*F. kuroshium*, *N. metavorans*, and *C. ficicola*) were not detected.

A positive correlation between the re-isolation rate (T1: *F. kuroshium*; T2: *N. metavorans*) and the distance from the inoculation site (0–40 cm) was confirmed by exponential function analysis (Figure S5). This upward trend implies new mycelial growth in these fungal species. Additionally, *N. metavorans* was re-isolated more frequently than *F. kuroshium* (Figure S5).

## 4. Discussion

In the present study, the pathogenicity of *C. ficicola* in the fig saplings was demonstrated by the development of FWD symptoms on the saplings after inoculation (Figure S1), as reported by Morita et al. [7]. Wilting of the leaves progressed rapidly, and after only 3–5 days, all leaves were desiccated and dead (Figure S2). To our knowledge, this is the first report on the use of dual isolates (*F. kuroshium* + *C. ficicola*) for FWD inoculation tests. A valuable observation was that the wilting speed of fig saplings in T4 was four days significantly faster than that in T3 (Figure 3), which is probably due to the synergistic action of the two fungal species. The xylem sap-conduction test indicated that the wood necrosis area of the dual-inoculated saplings was considerably wider compared to that of *C. ficicola* alone (Figure 5). These results suggest that the two fungal species can work together to negatively impact the physiology and biochemistry of the host fig trees. Thus, this study largely supports the hypothesis that dual inoculation with *F. kuroshium* and *C. ficicola* can induce early symptom development in fig saplings. However, the response in different fig varieties (e.g., ‘Horaishi’) as well as the specific mechanism of fungal invasion leading to sapling death needs further evaluation.

In California, USA, *F. kuroshium* associated with *E. kuroshio* was confirmed to be a pathogen in young avocado plants [20]. In Japan, *E. interjectus* inhabited fig orchards as a vector of *C. ficicola* [15,16], however, its associated mycangial fungus, *F. kuroshium* was harmless on its own in this study (Figure S2). This finding highlights a new concern; a combination of the ambrosia beetle and its ambrosia fungi may lead to FWD symptoms in the case of mass beetle attacks and decreased resistance in host trees. Therefore, *E. interjectus* should be monitored in its habitat to gain a better understanding of attack density. Moreover, further investigation into the full biotic and abiotic factors affecting host susceptibility and defense responses to *E. interjectus* is needed.

*Euwallacea interjectus* is responsible for weakening trees by gallery formation and the introduction of fungal pathogens (*C. ficicola*) [16], although the physiological changes in the trees are still unclear. A trigger possibility is that the first population of beetle attacks might confer stress on the host tree and promote fungal activity through the inner wall of the gallery as an entry point. Our study proposes the following possibility: infection with *C. ficicola*, probably being joined by *F. kuroshium*, inhibits the protective response of the tree, and increases the susceptibility to beetle attack by the next population.

**Figure 6:**
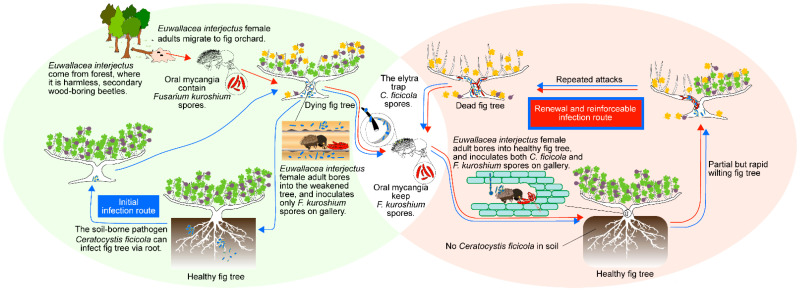
A schematic model for system changes of symbiosis among *Euwallacea interjectus* and its associated fungi in fig wilt disease.

Based on the present study, we suggest the following hypothesis to explain the dynamic wilting process of fig trees infested by *E. interjectus* in a fig orchard in Japan (Figure 6). Early reports indicate that the pathogen *C. ficicola* easily infects fig trees via roots embedded within the soil [8,32]. In this infection pattern, germicides in the soil can initially rescue the trees. Fig trees weakened by *C. ficicola* were found by *E. interjectus*, which originally inhabited the forest. Subsequently, *E. interjectus* began to bore into the weakened trees and reproduced the next generations in the fig orchard, inoculating the gallery with the mycangial fungus *F. kuroshium*. During this encounter between *E. interjectus* and fig trees, an additional relationship was established; *C. ficicola* was accompanied and carried on the elytra (fore-wing) of *E. interjectus* [15,16]. Thus, *C. ficicola* was used as “a weapon acquired by chance” for attacking healthy fig trees. In this novel infection pattern of *C. ficicola* to the trees via beetle, mycangial fungus surely plays a surprising role. Specifically, *F. kuroshium* can reinforce *C. ficicola* and thus bring fig trees to early symptom development, as demonstrated in this study. This may be one of the key factors suggested in FWD-endemic areas [6]. Finally, there is no doubt that trees will eventually die from partial but repeated *E. interjectus* attacks, while *C. ficicola* infection via the roots leads to overall sudden death with the expansion of brown xylem discoloration in the tree trunk near the ground [33]. Under renewed circumstances, sterilizing the soil no longer makes sense. Other integrated pest management strategies such as the control of the vector (*E. interjectus*) with insecticides [34] and natural enemies [35] should be considered.

According to our observations, 25 of the 36 FWD-infected saplings sprouted one or two shoots from the stem below the inoculation site (SS; Figures S1 and S2). Therefore, in the case of fungal infection via *E. interjectus*, the disease did not appear to progress to the roots. This phenomenon has not been reported in previous inoculation tests on fig saplings [7], although most of the roots of mature trees remain alive and can sprout in the orchard [33]. Further analysis of the invasion strategy of *C. ficicola* and *F. kuroshium* into fig trees below the inoculation site is necessary.

In conclusion, *F. kuroshium* and *N. metavorans* are not pathogenic to fig trees, and *F. kuroshium* is a potential causal agent of xylem dysfunction in trees, together with *C. ficicola*. It should be noted that *F. kuroshium* is a mycangial fungus outdoors and is symbiotically carried by *E. interjectus*. Through physiological stress, fig trees may be attacked by *E. interjectus*, but not *C. ficicola*, wherein *E. interjectus*-bound *F. kuroshium* causes FWD. Therefore, information on the “secondary” pathogenicity of *F. kuroshium* to fig trees will be helpful in developing methods to control FWD.

## Figures and Tables

**Table 1:** The inoculation of *Ficus carica* saplings with different isolates.

Treatment	Test Code	No. of Saplings	Inoculum	
			Source	Morphology
Wound inoculation	T1	18	*Fusarium kuroshium* ^a^	Hyphae + conidial masses
	T2	18	*Neocosmospora metavorans* ^b^	Hyphae
	T3	18	*Ceratocystis ficicola* ^c^	Hyphae + ascospore masses
	T4	18	*F. kuroshium + C. ficicola* ^d^	Hyphae + conidial/ascospore masses
Control	ST	18	Sterilized toothpicks ^e^	—
	CT	18	None ^f^	—

^a^ Isolated from the head of an adult female wild *E. interjectus* on 2 March 2018. ^b^ Isolated from the head of an adult female reared *E. interjectus* on 5 October 2017. ^c^ Isolated from soil surrounding the tree infected with FWD on 28 August 2009. ^d^ Detailed inoculation method, as shown in Figure 2. ^e^ Wounded control without fungal inoculum. ^f^ Non-wounded control.

## Data Availability

Data are contained within the article and Supplementary Materials.

## Supplementary Materials

The following supporting information can be downloaded at https://www.mdpi.com/article/10.3390/microorganisms10101912/s1, Figure S1: A typical wilt symptom in fig saplings inoculated with *Ceratocystis ficicola* (T3). Examples of T3-14 (NS), T3-09 (LW, BD), and T3-10 (SS) are shown. NS: No apparent difference was observed compared to the non-inoculated saplings; LW: Some leaves began to droop and wilt 12 days after inoculation; BD: All leaves became brown 15 days after inoculation; SS: One shoot sprouted (red arrow) below the inoculation site 9 days after death of the stem. Figure S2: Symptom development in each fig sapling after inoculation. Open circle: Beginning of leaf dropping; Filled circle: Browning of all leaves and dead of stem; Double circle: Dead of stem and sprouting below the inoculation site; X: Xylem sap-conduction test; R: Re-isolation test of the inoculated fungi; N: No use for X and R. Figure S3: Crosscut stem surfaces of fig saplings that absorbed the acid fuchsin solution. Examples of T3-08 and T4-01 (Dead), T1-01 and T2-07 (No external symptoms), ST-02 (Wound control), and CT-01 (Non-wounded control) are shown. Figure S4: Typical examples (i.e., 5a, 5b, 5c, 5d, 5e, and 5f in Figure S3) of pink areas dyed with acid fuchsin solution and brown discoloration on cross stem sections 5 cm above the inoculation site. SAC: Xylem sap-conduction area; DIS: Xylem discoloration area; Dotted line and arrow: Inoculation site and insert direction. Figure S5: Curve fitting of the re-isolation rate of *Fusarium kuroshium* in T1 and *Neocosmospora metavorans* in T2. *F. kuroshium*: *y* = 3.2235*e*^0.0758*x*^, *R*^2^ = 0.878, *p* = 0.005 < 0.01; *N. metavorans*: *y* = 9.6928*e*^0.0552*x*^, *R*^2^ = 0.835, *p* = 0.011 < 0.05.
