# Supplementary material for: The Role of Mycangial Fungi Associated with Ambrosia Beetles (Euwallacea interjectus) in Fig Wilt Disease: Dual Inoculation of Fusarium kuroshium and Ceratocystis ficicola Can Bring Fig Saplings to Early Symptom Development
Source: Microorganisms. 2022 Sep 27;10(10):1912. doi: 10.3390/microorganisms10101912 (PMC9607347; doi:10.3390/microorganisms10101912)
Supplement: Supplementary file 1 [file microorganisms-10-01912-s001.zip › microorganisms-1912369-supplementary.pdf]

microorganisms

Supplementary materials

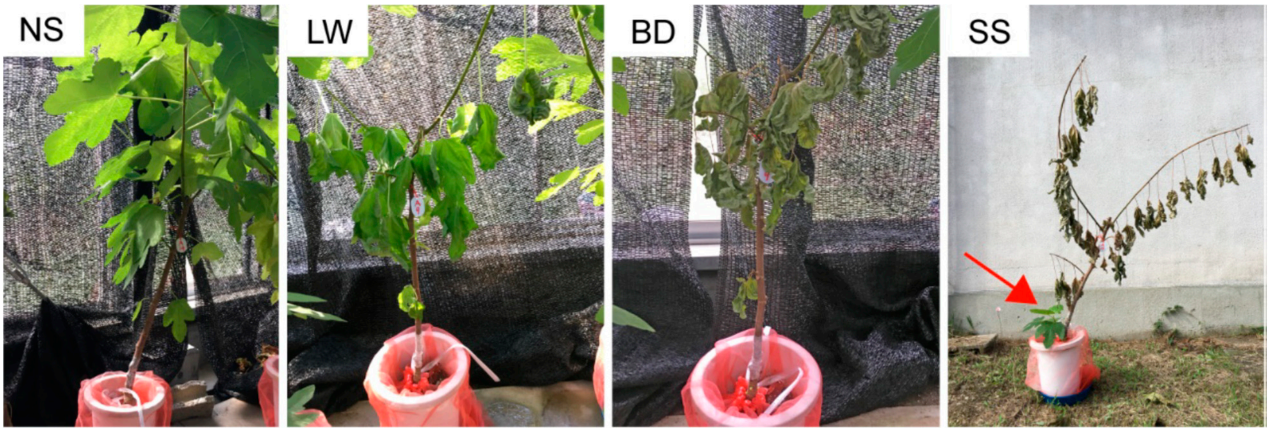

Figure S1. A typical wilt symptom in fig saplings inoculated with *Ceratocystis ficicola* (T3). Examples of T3-14 (NS), T3-09 (LW, BD), and T3-10 (SS) are shown. NS: No apparent difference was observed compared to the non-inoculated saplings; LW: Some leaves began to droop and wilt 12 days after inoculation; BD: All leaves became brown 15 days after inoculation; SS: One shoot sprouted (red arrow) below the inoculation site 9 days after death of the stem.

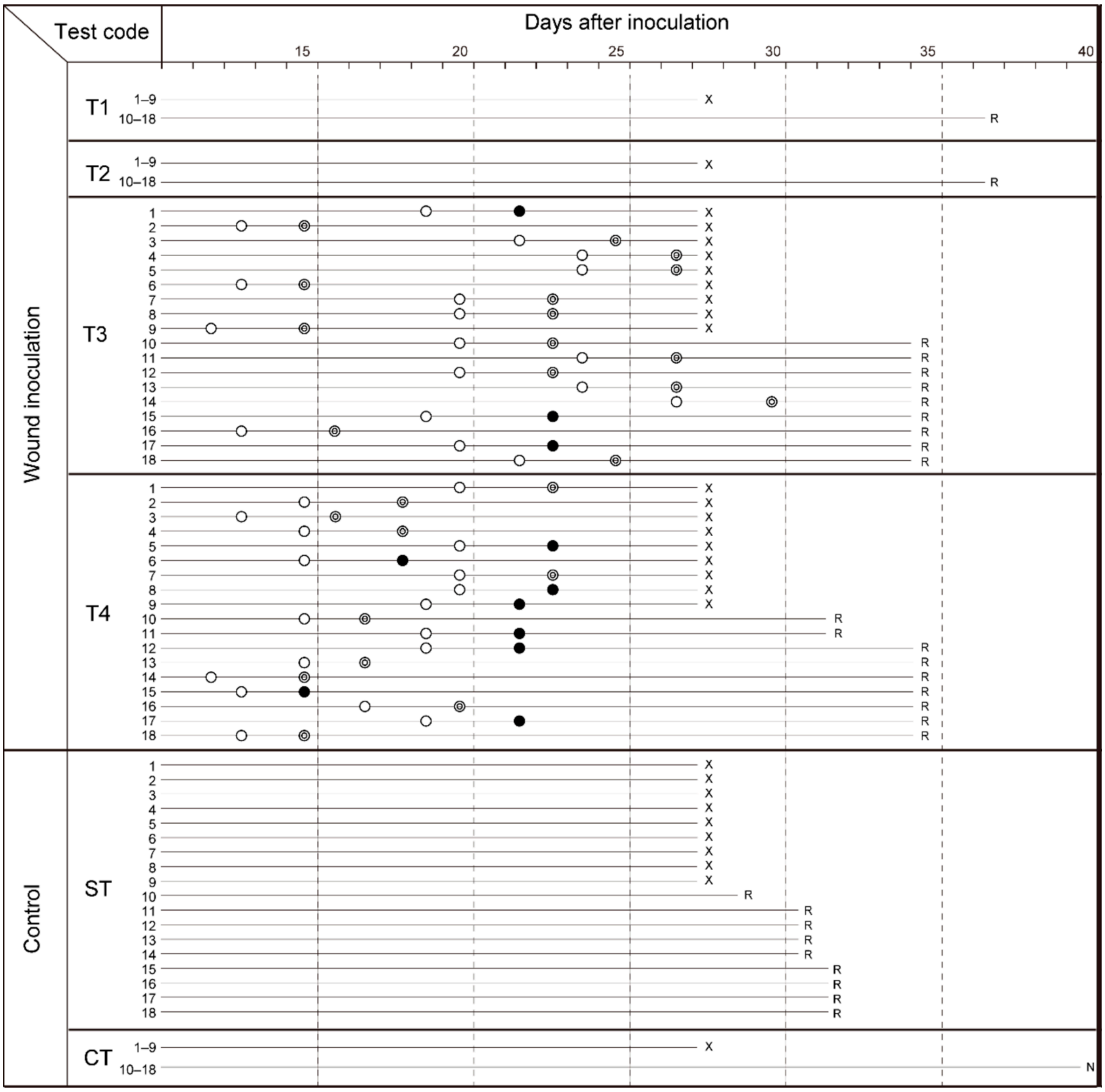

Figure S2. Symptom development in each fig sapling after inoculation. Open circle: Beginning.of leaf dropping; Filled circle: Browning of all leaves and dead of stem; Double circle: Dead of stem and sprouting below the inoculation site; X: Xylem sap-conduction test; R: Re-isolation test of the inoculated fungi; N: No use for X and R.

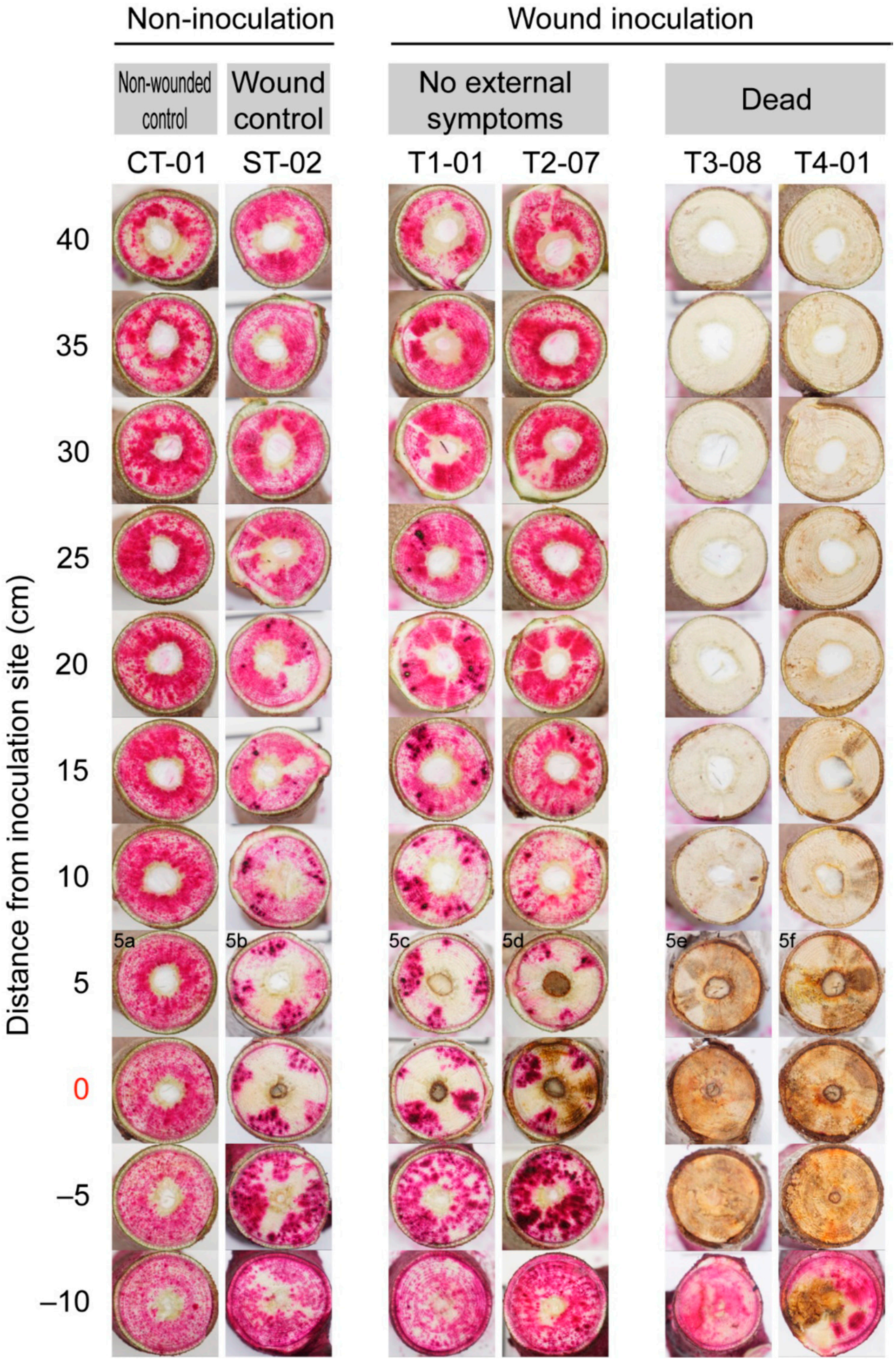

Figure S3. Crosscut stem surfaces of fig saplings that absorbed the acid fuchsin solution. Examples of T3-08 and T4-01 (Dead), T1-01 and T2-07 (No external symptoms), ST-02 (Wound control), and CT-01 (Non-wounded control) are shown.

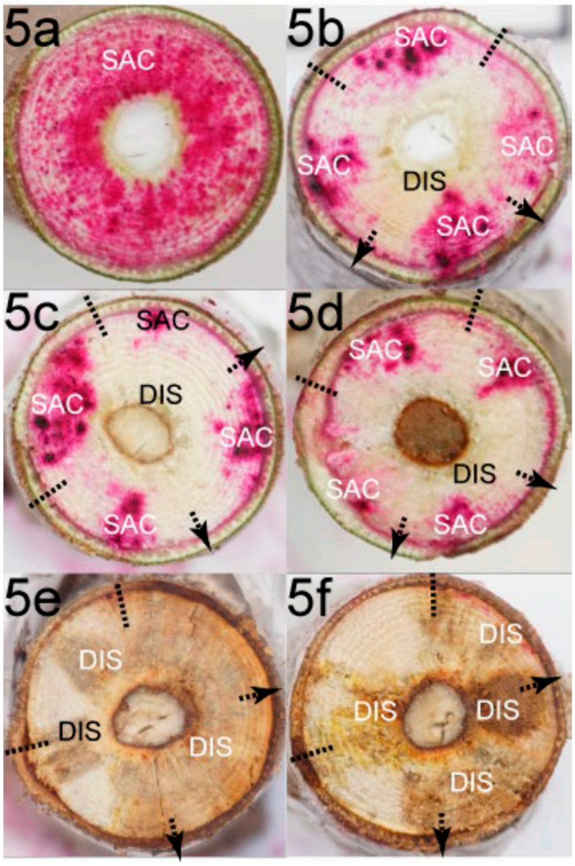

Figure S4. Typical examples (i.e., 5a, 5b, 5c, 5d, 5e, and 5f in Figure S3) of pink areas dyed with acid fuchsin solution and brown discoloration on cross stem sections 5 cm above the inoculation site. SAC: Xylem sap-conduction area; DIS: Xylem discoloration area; Dotted line and arrow: Inoculation site and insert direction.

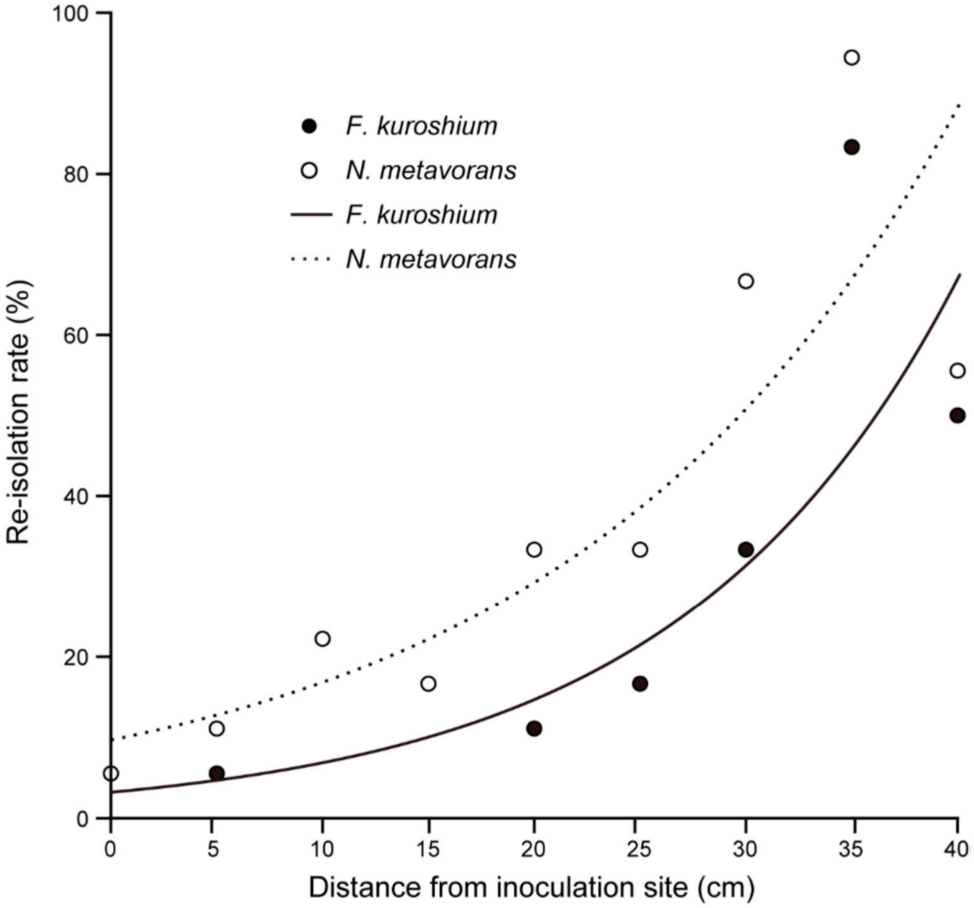

Figure S5. Curve fitting of the re-isolation rate of *Fusarium kuroshium* in T1 and *Neocosmospora metavorans* in T2. *F. kuroshium*: $y = 3.2235e^{0.0758x}$, $R^2 = 0.878$, $P = 0.005 < 0.01$; *N. metavorans*: $y = 9.6928e^{0.0552x}$, $R^2 = 0.835$, $P = 0.011 < 0.05$.
